# Supplementary material for: Successional Shifts in Soil C:N:P stoichiometry and its environmental controls during forest restoration in Huize County, Qujing, Yunnan Province
Source: PLoS One. 2026 Aug 21;21(8):e0356261. doi: 10.1371/journal.pone.0356261 (PMC13496410; doi:10.1371/journal.pone.0356261)
Supplement: Supplementary file 2 [file pone.0356261.s004.zip › Data and Code/RF/evergreen broadleaf forest/figures/PDP_N.P_C.P.pdf]

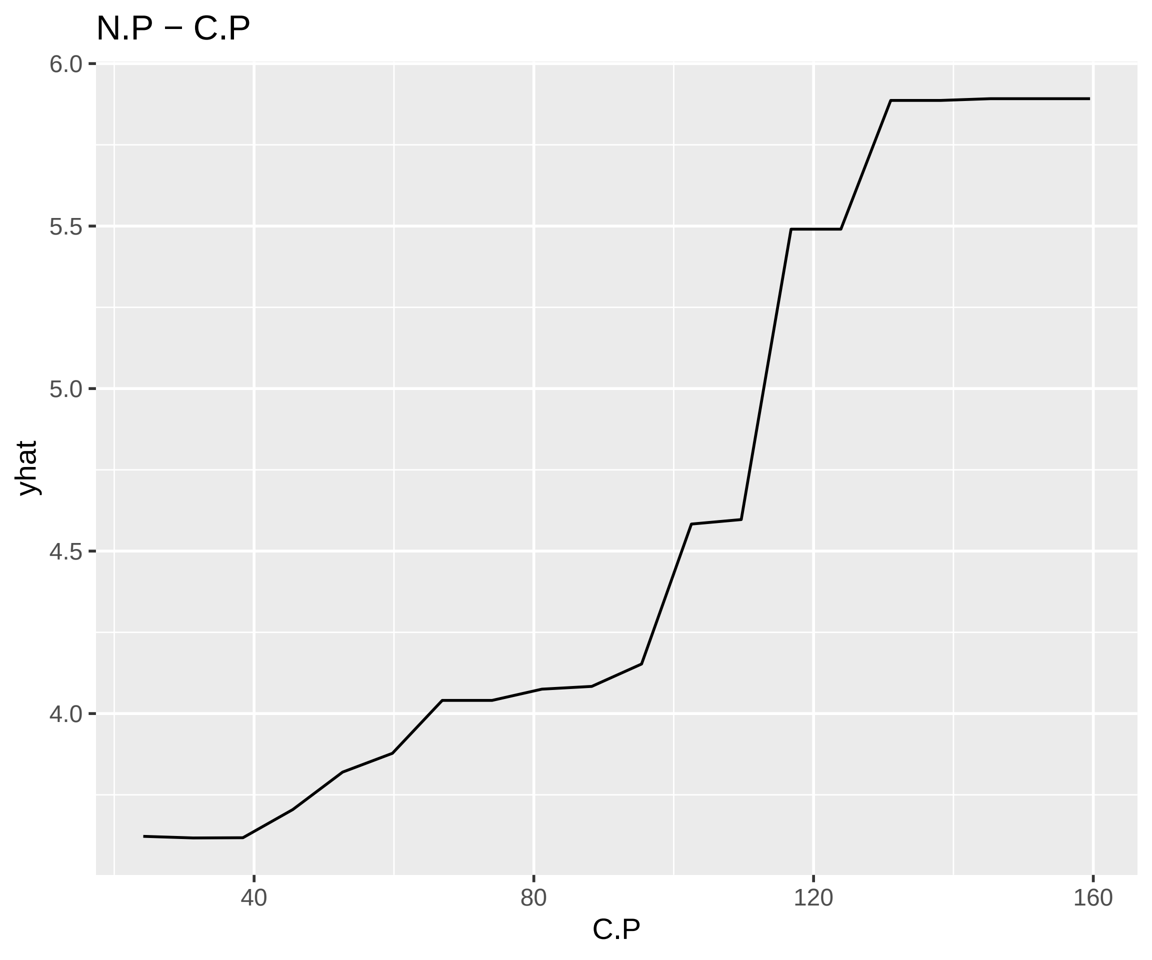
N.P − C.P
6.0
5.5
5.0
4.5
4.0
yhat
40
80
120
160
C.P
